# Chromatin remodelling comes into focus

**DOI:** 10.12688/f1000research.21933.1

**Published:** 2020-08-20

**Authors:** Ramasubramian Sundaramoorthy, Tom Owen-Hughes

**Affiliations:** 1 Centre for Gene Regulation and Expression, School of Life Sciences, University of Dundee, Dundee, Dundee, DD1 5EH, UK

**Keywords:** chromatin remodelling, SWI/SNF complex, nucleosome structure, INO80, SWR1, CHD1, SMARCA, BAF.

## Abstract

ATP-dependent chromatin remodelling enzymes are molecular machines that act to reconfigure the structure of nucleosomes. Until recently, little was known about the structure of these enzymes. Recent progress has revealed that their interaction with chromatin is dominated by ATPase domains that contact DNA at favoured locations on the nucleosome surface. Contacts with histones are limited but play important roles in modulating activity. The ATPase domains do not act in isolation but are flanked by diverse accessory domains and subunits. New structures indicate how these subunits are arranged in multi-subunit complexes providing a framework from which to understand how a common motor is applied to distinct functions.

## Introduction

In addition to packaging DNA within nuclei, chromatin provides a means of segmenting the genome into distinct chromatin states that ensure that transcription is regulated correctly in time and space ^1^. Conversion between chromatin states involves changes to chromatin at different levels, including post-translational modifications to histones and DNA, recruitment of chromatin-binding factors and direct changes to the structure of nucleosomes as a result of the action of chromatin remodelling ATPases.

ATP-dependent chromatin remodelling enzymes are molecular machines that act to reconfigure nucleosomes so as to enable gene regulation in response to developmental and environmental signals. Interest in this fundamental process has heightened with the finding that many subunits of these complexes are mutated at high frequencies in cancers and neurological disorders.

The involvement of chromatin remodelling ATPases in this process was first evident from the finding that mutations in the genes that encode components of these complexes affect processes such as mating type switching (SWI) and sucrose fermentation (SNF) in budding yeast ^2^. Many of the genes identified in these screens were found to associate as multi-subunit SWI/SNF complexes that had the ability to reconfigure chromatin ^3^. Subsequently, budding yeast were found to encode a related complex, remodels the structure of chromatin (RSC), in which many (but not all) subunits are paralogs of those found in SWI/SNF ^4^. Multicellular organisms also encode related complexes. In *Drosophila*, the Brahma complex falls within the trithorax group of developmental regulators ^5– 7^. In mammalian cells, three forms of SWI/SNF-related complexes have been identified ^6– 8^ ( Table 1). Homozygous loss of genes encoding most subunits results in early developmental defects; in humans, components of the complexes are frequently mutated in cancers ^9^. More recently, it has emerged that mutations to complex components are also detected in neurological disorders ^10^. Intriguingly, different subunits are found to be mutated both in cancers of different tissues and in neurological disorders.

**Table 1.** 

Yeast SWISNF	Yeast RSC	Human BAF	Human PBAF	
Snf2/Swi2	Sth1	SMARCA2/SAMRCA4	SMARCA4	SMARCA4
Swi1/Adr6	Rsc9	ARID1A /ARID1B	ARID2	GLTSCR1 / BICRAL1
Swi3	Rsc8	SMARCC1/SMARCC2	SMARCC1/SMARCC2	SMARCC1
Snf12/Swp73	Rsc6	SMARCD1/D2/D3	SMARCD1/D2/D3	SMARCD1
Snf5	Sfh1	SMARCB1	SMARCB1	
	Rsc2/4/58		PBRM /Brd7	BRD9
Swp82	Rsc7	DPF1/2/3	PHF10	DPF3
Arp7/9	Arp7/9	ACTL6A/ ACTL6B/β-Actin	BAF53A/BAF53B/β-Actin	BAF53A/BAF53B/β-Actin
	HTL1			
	Rsc3/30			
		SMARCE1	SMARCE1	

Biochemical characterisation of the complexes indicated that they can disrupt nucleosome structure ^3, 11^. Consistent with this, the complexes are linked to the maintenance of accessible chromatin structure at promoters in yeast and at enhancers in mammalian cells ^12– 18^. The complexes contain a catalytic subunit that is related to ancient ATP-dependent DNA translocases and that acts to drive DNA across the surface of nucleosomes. These specialised ATPase domains are found in an extended family of some 20 yeast and 40 human proteins that regulate DNA–protein interactions ^19^. In the context of SWI/SNF complexes, ATPase subunits act in the repositioning, destabilisation and dissociation of histones from DNA ^20^. Until recently, a structural framework on which the mechanism of action can be built has been lacking.

Here, we summarise recent insights into the structure of ATP-dependent remodelling enzymes. These show that remodelling enzymes share an ATPase module that acts on DNA within nucleosomes and that this motor domain is tuned to different purposes within distinct multi-subunit complexes.

## Snf2 ATPases: a motor domain for nucleosome reorganisation

**Figure 1.**
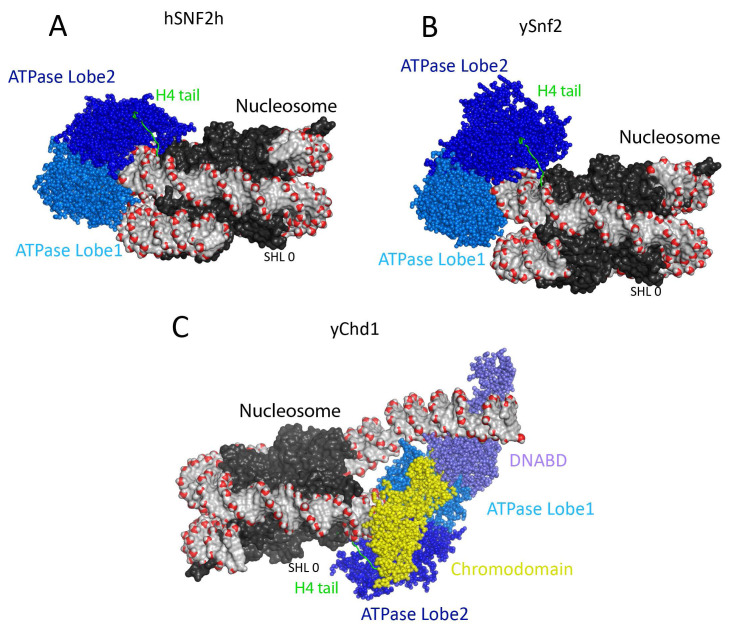
Structure of Snf2-related enzymes SNF2H, Snf2 and Chd1 bound to nucleosome. ( **A**) Structural model of *Saccharomyces cerevisiae* Snf2 ATPase domain fragment bound to a nucleosome at super helical location 2 (SHL ± 2) (PDB ID 5XOY) ^25^. The Snf2-related ATPase lobe1 and 2 are coloured in marine and blue spheres. The nucleosomal DNA is shown in surface representation, the backbone phosphate atom is highlighted in red sphere, and the dyad (SHL 0) of the nucleosome is marked. The histones are shown in black surface representation. The histone H4 tail that interacts with the lobe2 of ATPase domain is coloured green. ( **B**) Structural model of human ISWI remodeller SNF2H bound to nucleosome (PDB ID 6NE3) ^29^. The nucleosome and the ATPase lobes are presented in the same colours as in frame A. ( **C**) Structural model of Chd1 bound to nucleosomes. The structure of Chd1–nucleosome complex resolved at 4.5-Å resolution using cryogenic electron microscopy (PDB ID 6FTX) ^28^. The Chd1 ATPase shown in spheres is characterised by N-terminal tandem chromodomains and a C-terminal SANT-SLIDE–containing DNA-binding domain (DNABD). The Chd1 DNA-binding domain coloured in slate was found to be located at the edge of the nucleosome in the boundary between nucleosomal and linker DNA. The Chd1 ATPase lobes drawn in marine and blue are bound at the SHL 2 location distal to the linker DNA, and the chromodomains drawn in yellow at the SHL 1 location. Similar to the Snf2 and Snf2h, the histone H4 tail interacts with the ATPase lobe2. The dyad of the nucleosome is marked. Two turns of nucleosomal DNA prised from the surface of the histone octamer upon Chd1 binding.

The ATPase domains found in the yeast Snf2 protein are also present in an extended family of chromatin remodelling enzymes. Crystal structures of Rad54 and Chd1 proteins illustrated that each domain is made up of folds related to those found in bacterial RecA ^21– 23^. More recently, cryogenic electron microscopy (cryo-EM) has been used to obtain structures of yeast Snf2, human (SNF2H) and yeast ISWI (imitation SWI) proteins and yeast Chd1 proteins in complex with nucleosomes ^24– 29^ ( Figure 1). These studies show that each enzyme is capable of binding to nucleosomes two helical DNA turns away from their centres. Each of these enzymes engages with nucleosomes predominantly through contacts with DNA, and there are relatively few contacts with the histone components of nucleosomes. One exception observed in each structure is that the N-terminal region of histone H4 contacts the second ATPase domain. This region on the H4 tail is required for full activity of the SNF2H, Chd1 and Snf2 proteins and may act to ensure that full activity is reached only when the ATPase domains are correctly docked on nucleosomes. Higher-resolution studies provide enough detail to detect changes in the positioning of individual DNA bases in different nucleotide-bound states. Remarkably, binding in the presence of ADP results in the propagation of a distortion to DNA, predominantly on one strand, across some 55 base pairs of the octamer surface ^24^. As distinct distortions to DNA are observed in different nucleotide-bound states, it is possible to envision how co-ordinated small movements could drive DNA across the nucleosome surface ^12, 13^.

ATPase subunits are found within larger proteins. In the case of Chd1, chromodomains and a DNA-binding domain adjacent to the ATPase domain contribute to the nucleosome-bound state ( Figure 1C) and are sufficient to generate an enzyme active in chromatin remodelling. This does raise the question of why many remodelling ATPases are found as components of much larger multi-subunit complexes.

## The INO80 and SWR1 enzymes

**Figure 2.**
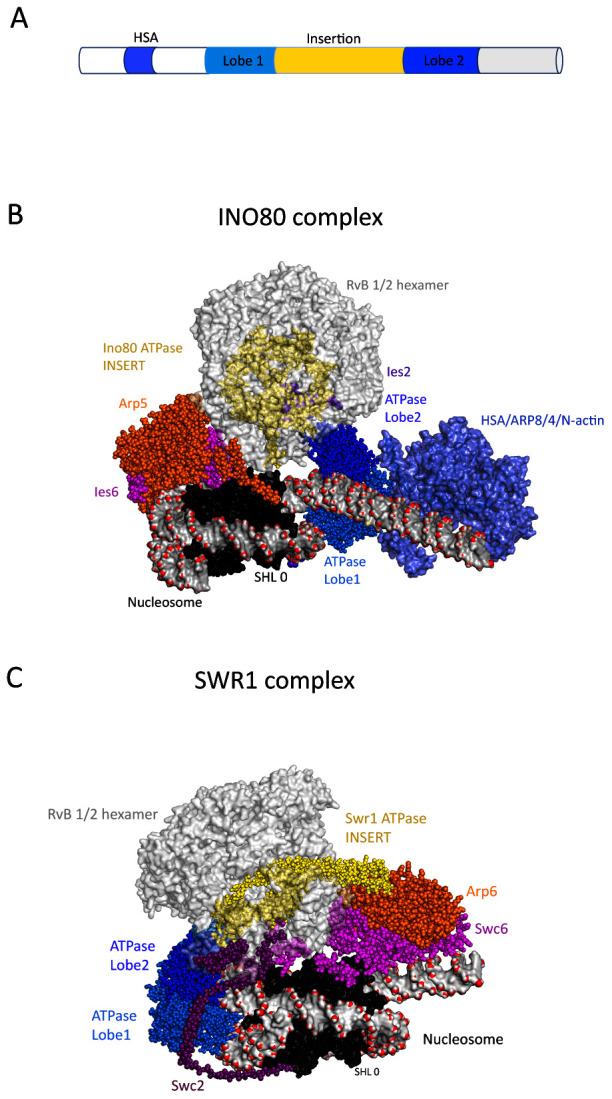
Structure of INO80 and SWR1 complexes. ( **A**) Schematic showing the signature ATPase subunit of the Ino80 and Swr1 remodellers. The ATPase is characterised by a split ATPase domain with a large insert region that associates with RuvB proteins and an N-terminal HSA domain that associates with ARP proteins. ( **B**) Structural model of INO80–nucleosome complex resolved at 4.3-Å resolution using cryogenic electron microscopy (cryo-EM) (PDB ID 6FML) ^32^. The subunits that form the INO80–nucleosome complex are labelled. The RuvB hexamer is shown in grey, and the Ino80 insert region that threads through RuvB hexamer in yellow. The nucleosome is shown in surface representation, and the DNA backbone phosphate atoms are highlighted as red spheres. The dyad of the nucleosome SHL 0 is labelled. The Ino80 HSA-Arp8-Actin-Arp4 module (PDB ID 5NBN) ^35^ is docked into EM density observed in this region ^33^ (coloured light blue). From this location, the Arp8-Actin-Arp4 module potentially interacts with nucleosomal linker DNA and these subunits do indeed cross-link with DNA in this region ^39^. Arp5 is shown in orange, Ies6 in pink and Ies6 purple. Ino80 ATPase lobe1 and 2 are coloured in marine and dark blue respectively. ( **C**) Structural model of SWR1–nucleosome complex resolved at 3.6-Å resolution using cryo-EM (PDB ID 6FML) ^31^. The RuvB hexamer is shown in light grey, and the Swr1 insert region that threads through RuvB hexamer in yellow sphere. This extended helical region protrudes through the RuvB hexamer and makes contact with the Arp6 subunit (orange). The Swc6 and Swc2 subunits are coloured pink and purple. The nucleosome and ATPase domains are represented as in frame B.

A first close-up view of multi-subunit remodelling ATPases came from the structural characterisation of the INO80 and SWR1 complexes ^31– 33^ ( Figure 2). Surprisingly, the ATPase domains of these complexes engage with nucleosomes at different locations. The Ino80 ATPase domains interact with DNA at the edge of the nucleosomes causing unwrapping of outer turns of DNA, whereas in Swr1 they interact at the off-centre site also observed for Snf2 and Chd1. Despite these differences, the Swr1 and Ino80 proteins share distinctive large insertion regions separating the two ATPase domains. In both cases, the insert regions are intertwined with a hetero-hexamer of RuvB proteins, a second type of conserved ATPase belonging to the AAA+ group that are found in both INO80 and SWR1 complexes. The Ino80 and Swr1 insert domains appear to represent a specific adaption of the Snf2-related ATPase domains to function in concert with hetero-hexameric RuvB-related proteins. Mysteriously, ATP hydrolysis by the RuvB proteins is not required for Swr1 histone exchange activity; as a result, they have been proposed to function as a scaffold ^31^. The INO80 and SWR1 complexes also contain hetero-dimers of proteins that contain actin folds. These include the Arp5/Ies6 hetero-dimer in the INO80 complex and the Arp6/Swc6 hetero-dimer in the SWR1 complex. These dimers interact on the side of the nucleosome opposite to that occupied by the ATPase domains. In the case of the SWR1 complex, the Arp6/Swc6 interacts with outer turns of nucleosomal DNA at super-helical location (SHL) 6 and histone H2A/H2B dimer surface and generates an unwrapping of one turn of nucleosomal DNA ^14, 15^. In the case of Arp5/Ies6, the interaction is internal to the nucleosome. INO80 and SWR1 also contain a second grouping of actin fold proteins: the Arp8/Arp4/actin module. This module has been studied in isolation and is known to interact with a helicase-SANT-associated (HSA) domain present N-terminal to the ATPase domains of many remodelling ATPases where it acts as a linker DNA-binding module that regulates activity ^16– 19^. Density for the Arp8/Ar4/actin module is not observed in one structure ^32^, but weak density is observed in the other ^33^ ( Figure 2B). From this location, the module is well placed to interact with extranucleosomal linker DNA. Consistent with this, this region cross-links to linker DNA and regulates the coupling between ATP-hydrolysis and nucleosome repositioning ^39^.

In summary, while the INO80 and SWR1 complexes share insert regions that mediate interactions with RuvB hetero-hexamers, they engage with nucleosomes in different orientations. The two complexes also have distinct biochemical activities. INO80 is capable of repositioning nucleosomes ^20, 21^, whereas SWR1 directs replacement of histone H2A/H2B dimers with the variant histone H2A.Z/H2B dimers ^42^. The molecular mechanisms underlying these distinct outcomes remain to be determined.

## SWI/SNF complexes: multi-tools for chromatin remodelling

**Figure 3.**
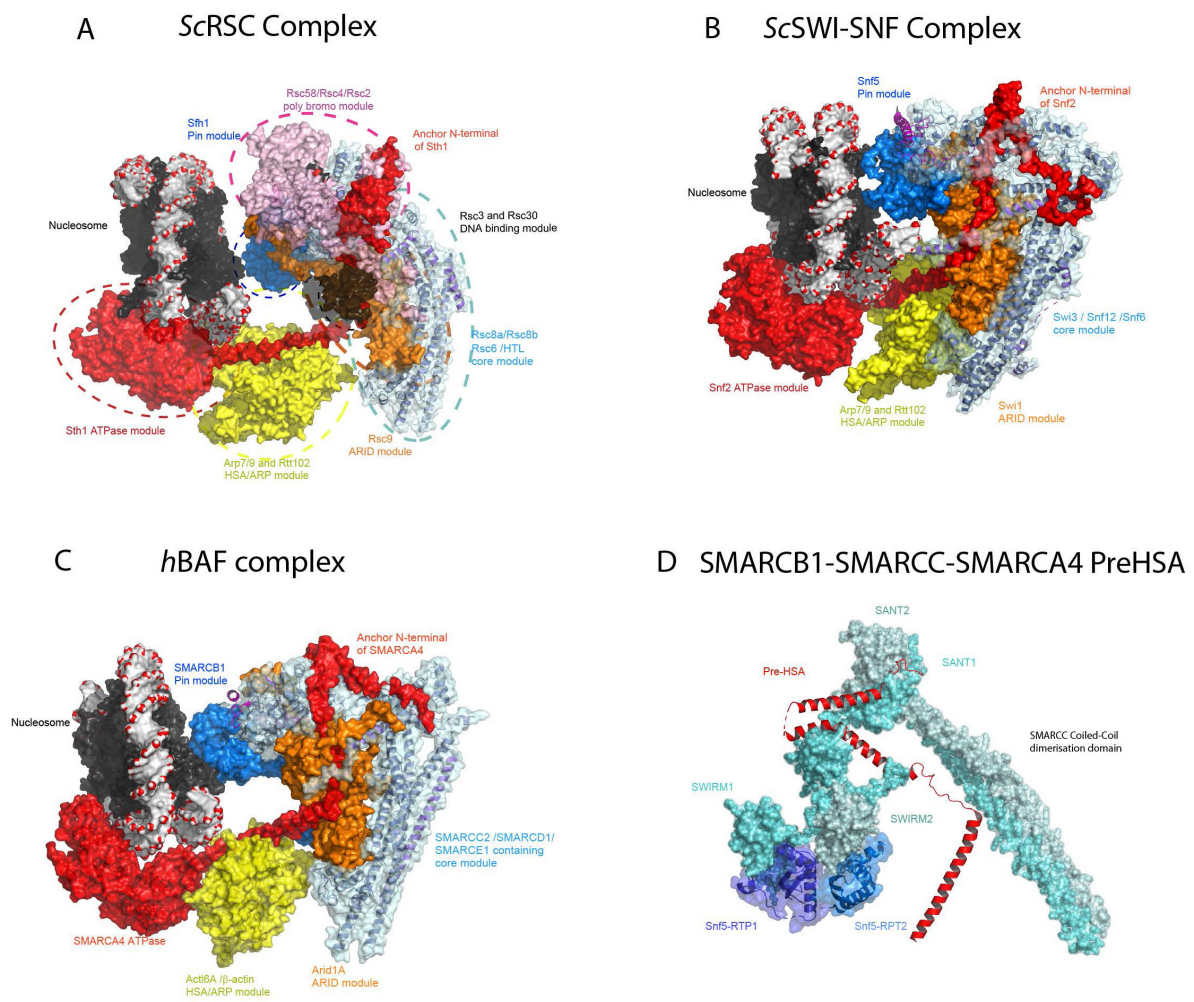
Structure of the RSC and human SWI/SNF (BAF) complex bound to a nucleosome. ( **A**) The structure of the RSC–nucleosome complex (PDB ID 6K15, 6KW3, 6KW4) ^46^. The structure can be considered a core or hub module made up principally of the Rsc8 and Rsc6 subunits which are coloured teal. From this, additional modules with distinct functionality extend in different directions: the armadillo repeat-containing Rsc9 is shown in orange; Sfh1 which contacts the nucleosome lateral surface, the pin module, is shown in marine; the two tandem bromodomain-containing subunits Rsc2 and Rsc4, together with Rsc58, are coloured pink; the main ATPase Sth1 is coloured red; and Arp7-Rtt012-Arp9 are coloured yellow. The bound nucleosome is shown in similar representation as in Figure 1 and Figure 2. ( **B**) The structure of the SWI/SNF chromatin remodelling complex PDB 6UXW ^48^. Homologous subunits ( Table 1) are coloured similarly to those in RSC. The Swi3, Snf12 and Snf6 constituents of the core module are coloured in teal, the armadillo repeat-containing Swi1 protein in orange, the Arp module in yellow, the Snf5 pin module in marine, the Snf2 ATPase in red and the nucleosome as in frame A. ( **C**) The structure of Arid1A-containing human BAF complex (PDB 6LTH, 6LTJ) ^47^. The complex has a geometry similar to that of the yeast RSC and SWI/SNF complexes. The core of the BAF complex is composed of the SMARCC2 dimer, the HMG domain-containing SMARCE1 protein and the SWIB domain-containing SMARCD1 protein and is shown in teal. The SWIRM domain of the SMARCC2 dimer mediates interaction with the tandem RPT domain of SMARCB1 protein shown in marine. The nucleosome acidic patch is contacted by the SMACB1 C-terminal region, and the pin module is similar to that observed in the RSC and SWI/SNF–nucleosome complexes. SMARCA4 ATPase is shown in red bound to the nucleosome at SHL 2 location and the N-terminal region of ATPase and the pre-HSA domain anchored onto the core of the complex. The Arid1A armadillo repeat domain drawn in orange occupies the central cavity formed by the L-shaped SMARCC2 dimer. The ARP module drawn in yellow surface constitutes the Actl6A/β-actin dimer associated with the ATPase HSA domain and forms a bridge between the SMARCA4 ATPase and the core module. ( **D**) Expanded view of SMARCC dimer coloured cyan and dull blue. SMARCC2 has N-terminal SWIRM and SANT domains. SMARCB1 tandem repeat domains (RPT1 and RPT2), shown in blue and marine, interact with each of the SWIRM domains from the dimer of SMARCC2. Similarly, the SANT domain clamps the N-terminal region of ATPase SMARCA4 shown in cartoon representation and coloured in red.

As with the INO80 and SWR1 complexes, cryo-EM has been applied to determine the structures of the yeast SWI/SNF and RSC complexes. Initially, lower-resolution structures indicated that these complexes are globular with a C-shaped central cavity of appropriate dimensions to accommodate a nucleosome ^22– 25^. Very recent higher-resolution structures of budding yeast RSC and SWI/SNF and human BAF complexes reveal a distinct organisation in which the Rsc8, Swi3 or human SMARCC proteins form a dimeric hub at the core of the complex ^46– 50^ ( Figure 3A–D). Yeast Rsc8, Swi3 and the human SMARCC1 and SMARCC2 proteins all contain N-terminal SWIRM domains, Zinc finger-binding modules, a SANT domain and C-terminal dimerisation domains. Within the complexes, the long dimerisation helices interact in a parallel orientation reminiscent of the Fos/Jun dimerisation module ^51^ ( Figure 3D). Furthermore, the N-terminal SWIRM domains are not arranged symmetrically but adopt distinct conformations ( Figure 3D). This asymmetry is likely imposed by the interaction of the two SWIRM domains with tandem repeats within the Sfh1/Snf5/SMARCB1 protein ( Figure 3D). The Rsc8/Swi3/SMARCC dimerisation interface is also contacted and likely stabilised by Rsc6/Snf12/SMARCD1 and the N-terminal region of the ATPase subunit Sth1/Snf2/SMARCA4. The core formed by the Rsc8/Swi3/SMARCC dimers and Rsc6/Snf12/SMARCD1 is conserved from yeast to humans and these subunits are present within the three major forms of SWI/SNF-related complex present in humans ( Table 1). This core likely serves as a platform from which accessory modules adapt the complex for distinct functions. Within the RSC, SWI/SNF and BAF complexes, five distinct modules are appended to the core ( Figure 3):

i) Adjacent to the Rsc8 dimerisation helices, the armadillo repeats of the Rsc9/Swi1/ARID1A protein are visible. The ARID1A and ARID2 proteins are distinguishing features of the BAF and PBAF forms of mammalian complex positioned some distance from the ATPase domains and likely provide as-yet-uncharacterised complex-specific functionality.
ii) The tandem bromodomain-containing Rsc2 and Rsc4 proteins are located close to the C-terminus of Rsc8. In mammalian PBAF complex, the polybromo protein PBRM1 is likely to interact at a similar location.
iii) In between these regions, the HSA domain of Sth1/Snf2/SMARCA4 protrudes *en route* to the motor domains which engage with the nucleosome in an orientation similar to that observed with the isolated Snf2 protein. The HSA domain interacts with the actin fold subunits Arp7 and Arp9 and, together with Rtt102, has been crystallised independently ^36^. This HSA-ARP module links the ATPase domains to the central core based around the Rsc8 dimer. This module is shared in SWI/SNF, RSC, INO80 and SWR1 complexes ^16– 19^.
iv) The nucleosomal linker DNA extends back towards the Rsc8 core and may make contact with a DNA-binding domain which, at present, is poorly resolved but likely to be composed of the Rsc3 and Rsc30 subunits.
v) The Sfh1/Snf5/SMARCB1 protein C-terminus extends back forming a distinct nucleosome-binding module located such that it is placed to interact with the acidic patch region on the lateral surface of the nucleosome. This contact is shared in SWI/SNF and RSC complexes that also have the ability to both reposition and evict nucleosomes but is not present in SNF2H and Chd1 complexes which only reposition nucleosomes. Furthermore, mutation of the C-terminal region of Sfh1 that interacts with the acidic patch specifically decreases the ability of RSC complexes to evict nucleosomes with little effect on ATPase activity ^46^. This contact is conserved in human forms of SWI/SNF complex ^47^ and mutations in this region observed in patients with Coffin–Siris syndrome also affect the ability of complexes to reconfigure nucleosomes ^52^. It is possible that contact with this region confers specificity for histone variants ^53^.
vi) The structures of nucleosome-bound complexes are in all cases relatively low and combined with higher-resolution structures of nucleosome-free complexes. This means that it is not possible to determine whether detail observed in the higher-resolution structures of the Snf2 ATPase fragment bound to a nucleosome, including the location of the histone H4 tail, hold true in the context of nucleosome-bound complexes.

Overall, the structure can be considered a central hub from which modules representing sites of association for ARID-containing proteins, multi-bromodomain–containing subunits, the ATPase domains, a linker DNA-binding domain and a module that interacts with the lateral surface of the nucleosome are projected in different directions ( Figure 3A–C). The need to accommodate these modules, each likely to be associated with distinct functionalities, goes some way to explaining the larger size of SWI/SNF complexes.

## Mutations to SWI/SNF components in human disease

The major features of the RSC nucleosome structure are conserved in the yeast SWI/SNF complex and many of the subunits have equivalents in human forms of SWI/SNF complex ( Table 1). A major difference in humans is that there are two genes most similar to Rsc8 in humans, *SMARCC1* and *SMARCC2*. These can be present as either hetero- or homo-dimers within SWI/SNF complexes, but the published structure contains only SMARCC2.

Many features of the modular organisation of the complex are consistent with previous observations. For example, SWI/SNF-related complexes remain substantially intact following loss of most subunits ^7, 26– 28^. This is consistent with the idea that individual modules are largely independent. The exception is the core of the complex which, through acting as a scaffold for association of many components, plays a more significant role in complex integrity. Consistent with this, perturbing SMARCC1 and SMACC2 levels results in substantial degradation of SWI/SNF complexes ^7, 29, 30^. Similarly, loss of all SMARCD isoforms (equivalent to Rsc6) results in disruption of core complexes and recovery of a residual core module ^7^. Subunits that are not assembled correctly are subject to ubiquitinylation and proteasome-mediated degradation ^29, 30^. Differences in the potency of this surveillance system may explain some differences in the effects of deleting subunits in specific cell types. For example, the peripheral association of the Sfh1/Snf5/SMARCAB1 subunits would suggest that these are not required for complex integrity. Consistent with this, SWI/SNF complexes remain largely intact following deletion of the Snf5 subunit with only Swp82 and Taf14 dissociating ^26, 27^. Deletion of the human homolog *SMARCB1* does not severely compromise complex integrity in HEK293T cells ^7^ but does in rhabdoid cell lines ^17^. Thus, subtle effects on subunit associations may have different effects on complex integrity in different tissue types. The partial or complete dissociation of complexes following loss of subunits is, of course, relevant to diseases in which these subunits are lost. However, it does not necessarily inform on the pathway by which complexes are assembled which may be distinct. To characterise how complexes assemble requires study of the order in which nascently translated peptides associate, as observed for assembly of SAGA complexes ^59^.

**Figure 4.**
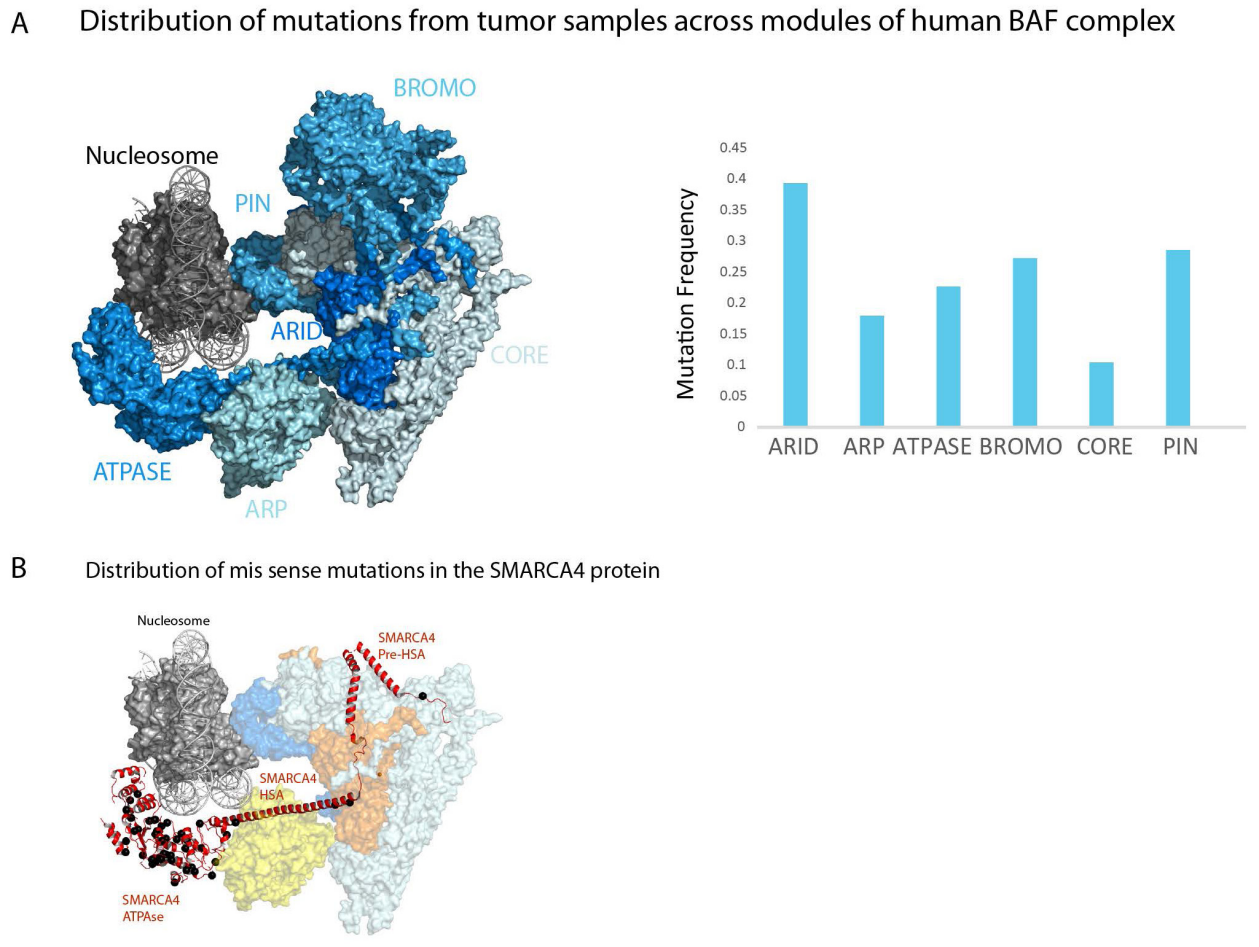
Depiction of different modules of BAF/PBAF complex and mutation rates. ( **A**) A schematic representation of the major modules of human BAF and PBAF remodelling enzymes. Placement of modules is based on structures shown in Figure 3; the subunits included in each module are listed in Table 2. Note that the bromo module is not present in BAF forms of complex. Subunits are coloured by mutation frequency which is also represented as a graph. Mutation frequency is the sum of truncating and missense mutations in the coding regions of genes encoding each subunit and recovered from cbioportal ^61^ divided by the coding length in base pairs. Truncating mutations and mutations annotated as likely to be oncogenic are more highly enriched in peripheral modules and depleted from the core region ( Table 2). ( **B**) Sites of missense mutations within SMARCA4 (red) are shown in black. Mutations are greatly enriched in the ATPase domains in comparison with the HSA domain that interacts with ARP proteins or the N-terminal region which is folded into the core region. Mutated sites obtained from non-redundant studies cohort at cbioportal ^61^.

It is estimated that 20% of all human cancers contain a mutation to at least one subunit of one form of SWI/SNF complex ^9^. However, the ARID1A, PBRM1 and SMARCA4 subunits are mutated at highest frequencies and these are mutated at much higher rates in tumours of some tissues in comparison with others ( Table 2). It is notable that the SMARCC1 and SMARCC2 subunits that form the core of the complex and are essential for its integrity ^58^ are mutated at substantially lower rates ( Table 2 and Figure 4A). It is possible that mutations that severely compromise complex integrity are disfavoured. Instead, mutations to a subset of subunits that affect one aspect of SWI/SNF functionality are those mutated at high rates ( Table 2 and Figure 4A). The ARID1A component which shares armadillo repeats with Rsc9 and Swi1 represents a discrete region adjacent to the core of the complex which remains intact following loss of ARID1A ^28, 31^. Similarly, the PBRM1 component contains six bromodomains and is likely related to the Rsc2, Rsc4 and Rsc58 subunits which occupy a location at one extremity of the core region. Deletion of PBRM1 does not affect association of other subunits ^7, 28^. Mutations to SMARCB1 which is related to the yeast *SFH1* and *SNF5* genes are relatively rare but debilitating as they drive malignant rhabdoid tumours ^60^. The relatively low frequency of SMARCB1 mutations may be related to the fact it has no obvious partially redundant paralogs and is present in two forms of human SWI/SNF-related complex. SMARCA4 is mutated at relatively high frequency, but unlike other subunits, these mutations are predominantly missense mutations clustering to the ATPase domains and appear to act in a dominant fashion ^16^. In contrast, the high frequency of mutations in ATPase domains, the HSA and N-terminal region of SMARCA4 is mutated at relatively low frequency ( Figure 4B). As a result, the distribution of mutation within the SMARCA4 module re-enforces the notion that loss of specific aspects of the function of the complexes—in this case, ATPase activity—drives cancer, rather than mutations that affect the core region and destabilise the entire complex.

**Table 2.** 

Gene ID	Module ^1^	Missense-Mutation	Truncating- mutations ^1^	Mutations/bp ^2^	Predicted oncogenic ^3^
Arid1A	ARID	1110	2231	0.487381473	231
ARID2	ARID	878	648	0.277202543	74
Arid1B		960	334	0.192903995	24
SAMRAC4	ATPASE	1306	335	0.332119004	32
SMARCA2	ATPASE	489	74	0.11802935	1
SMARCC1	CORE	257	61	0.095927602	NA
SMARCC2	CORE	337	93	0.118066996	NA
SMARCD1	CORE	160	42	0.130744337	NA
SMARCD2	CORE	119	30	0.093534212	NA
SMARCD3	CORE	103	16	0.082125604	NA
SMARCE1	CORE	95	24	0.096512571	NA
SMARCB1	PIN	233	97	0.285714286	15
ACTL6A	ARP	105	31	0.105672106	NA
ACTB	ARP	275	23	0.264888889	NA
PBRM1	BROMO	661	721	0.272745214	35
DPF1		99	31	0.114035088	NA
DPF2		136	35	0.145780051	NA
DPF3		139	31	0.149911817	NA
PHF10		100	54	0.103078983	NA
BRD7		147	91	0.121863799	NA

^1^This indicates the module the protein product of gene is assigned to in Figure 4

^2^This is the sum of missense and truncating mutations identified from cBioPortal non-redundant studies divided by the coding sequence length in base pairs.

^3^This indicates number of mutations associated with annotation indicating a potential oncogenic function.

This first wave of structures provides a first insight into the layout of different classes of chromatin remodelling enzyme. Though spectacular, substantial proportions of many subunits are not defined, meaning that it is not possible to assign functions to the bromodomain and armadillo repeat-containing subunits that define different forms of complex. In addition, the current structures represent snapshots of moving machines. To build a complete picture of their function will require views of different stages of the reactions they drive. It will also be critical to determine where and how their activity is regulated by contacts with interaction partners. Nonetheless, insight into the complexes is proceeding at a dramatic pace and provides a structural framework encompassing many of the subunits and domains present.

## Abbreviations

HSA, helicase-SANT-associated; Ies, inositol eighty subunit; INO80, INOsitol requiring; RSC, remodels the structure of chromatin; SMARCC, SWI/SNF-related matrix-associated actin-dependent regulator of chromatin; SWI/SNF, mating type SWItching/sucrose non-fermenting; SWR1, SWI-related 1; cryo-EM, cryogenic electron microscopy.
